# Pinewood nematode-associated bacteria contribute to oxidative stress resistance of *Bursaphelenchus xylophilus*

**DOI:** 10.1186/1471-2180-13-299

**Published:** 2013-12-23

**Authors:** Cláudia S L Vicente, Yoriko Ikuyo, Manuel Mota, Koichi Hasegawa

**Affiliations:** 1 Department of Environmental Biology, College of Bioscience & Biotechnology, Chubu University, 1200 Matsumoto, Kasugai, Aichi 487-8501, Japan; 2 ICAAM - Instituto de Ciências Agrárias e Ambientais Mediterrânicas, Departamento de Biologia, Universidade de Évora, Núcleo da Mitra, Ap. 94, Évora 7002-554, Portugal; 3 INIAV/Unidade Estratégica de Investigação e Serviços de Sistemas Agrários e Florestais e Sanidade Vegetal Av. da República, Quinta do Marquês, Oeiras 2784-159, Portugal

**Keywords:** *Bursaphelenchus xylophilus*, Catalase, Oxidative stress, Pine wilt disease

## Abstract

**Background:**

Pine wilt disease (PWD) caused by the pinewood nematode *Bursaphelenchus xylophilus* is one of the most serious forest diseases in the world. The role of *B. xylophilus*-associated bacteria in PWD and their interaction with the nematode, have recently been under substantial investigation. Several studies report a potential contribution of the bacteria for the PWD development, either as a helper to enhance the pathogenicity of the nematode or as a pathogenic agent expressing interesting traits related to lifestyle host-adaptation.

**Results:**

We investigated the nematode-bacteria interaction under a severe oxidative stress (OS) condition using a pro-oxidant hydrogen peroxide and explored the adhesion ability of these bacteria to the cuticle surface of the nematodes. Our results clearly demonstrated a beneficial effect of the *Serratia* spp. (isolates LCN-4, LCN-16 and PWN-146) to *B. xylophilus* under the OS condition. *Serratia* spp. was found to be extremely OS-resistant, and promote survival of *B. xylophilus* and down-regulate two *B. xylophilus* catalase genes (*Bxy-ctl-1* and *Bxy-ctl-2*). In addition, we show that the virulent isolate (Ka4) of *B. xylophilus* survives better than the avirulent (C14-5) isolate under the OS condition. The bacterial effect was transverse for both *B. xylophilus* isolates. We could not observe a strong and specific adhesion of these bacteria on the *B. xylophilus* cuticle surface.

**Conclusions:**

We report, for the first time, that *B. xylophilus* associated bacteria may assist the nematode opportunistically in the disease, and that a virulent *B. xylophilus* isolate displayed a higher tolerance towards the OS conditions than an avirulent isolate.

## Background

Pine wilt disease (PWD), caused by the migratory plant parasitic nematode *Bursaphelenchus xylophilus* (the pinewood nematode, PWN), is one of the most serious global forest diseases [1]. *B. xylophilus* and its vector beetles are listed as worldwide quarantine pests [2,3]. Under laboratory conditions, *B. xylophilus* has been reported to be sufficient for PWD development [4]. However, because of their ubiquitous existence in the PWD environments, some bacteria have also been thought to be involved in the disease development. For example, some *B. xylophilus-*associated bacteria are beneficial to *B. xylophilus* growth and reproduction [5], and others have been suggested or demonstrated to produce interesting bacterial traits that may contribute to *B. xylophilus* pathogenic potential and, ultimately, to PWD development [6-9].

Plant oxidative burst comprises in the production of reactive oxygen species (ROS) as a result of the interaction between plant cell receptors and pathogen-elicitors immediately after pathogen invasion [10-12]. Being relatively stable and permeable to the cell membrane, hydrogen peroxide (H_2_O_2_) is the most predominant ROS in plant oxidative burst [13,14]. In addition, H_2_O_2_ leads to the formation of the radical OH, which is extremely reactive and for which there is no scavenging system [15]. H_2_O_2_ was found to be transversal in different plant-pathogen systems, being a fundamental diffusible signal in plant resistance to pathogens (i.e. involved in cell-wall reinforcement or induction of defence-related genes in healthy adjacent tissues) [16].

Plant pathogens have evolved different evasion features to protect themselves against plant oxidative stress (OS) [17]. Bacterial defences include production of extracellular polysaccharides (EPS) coating and periplasmic catalases, and cytoplasmic catalase and superoxide dismutases (SOD) to counteract ROS before and after entering bacterial cells [18,19]. Other factors are related to the production of polyesters, poly-(3-hydroxyalkanoate) (PHA) also known as protective molecules [18], or phytotoxins (i.e. coronatine in *Pseudomonas syringae*) that are able to manipulate or down regulate plant-defences for bacteria successful establishment [20]. In plant- or animal-parasitic nematodes, antioxidant enzymes have been found to be the important weapons against oxidative stress of their plant- or animal-hosts [21]. Molinari [22] detected different antioxidant enzymes in *Meloidogyne incognita*, *M. hapla*, *Globodera rostochiensis*, *G. pallida*, *Heterodera schachtii*, *H. carotae*, and *Xiphinema index* and their relationship with life stages. Robertson *et al*. [23] and Jones *et al*. [24] have studied, the role of host ROS breakdown by peroxiredoxins (PXN) and glutathione peroxidases (GXP) in *G. rostochiensis*, respectively. Bellafiore *et al*. [25] reported the presence of several detoxifying enzymes, in particular glutathione S-transferases (GST), in the secretome of *M. incognita* as means of controlling the global oxidative status and potential nematode virulence.

*Pinus thunbergii*[26] and *P. pinaster*[27] are the *B. xylophilus*-susceptible pine trees found in Japan and Europe (Portugal) to respectively, respond to a strong oxidative burst in the earliest stages of nematode invasion. Most likely, *B. xylophilus* has developed an efficient antioxidant system to diminish the deleterious effects of oxidative burst in their invasion and colonization [28], as well as other plant parasitic nematodes [29]. Our study aimed to understand the tolerance of the *B. xylophilus*-associated bacteria under the OS condition and its interaction with the nematode. Also, we explored the bacterial attachment to the nematode cuticle for dissemination purposes.

## Results

### *B. xylophilus* and associated *Serratia* in stress conditions

**Figure 1:**
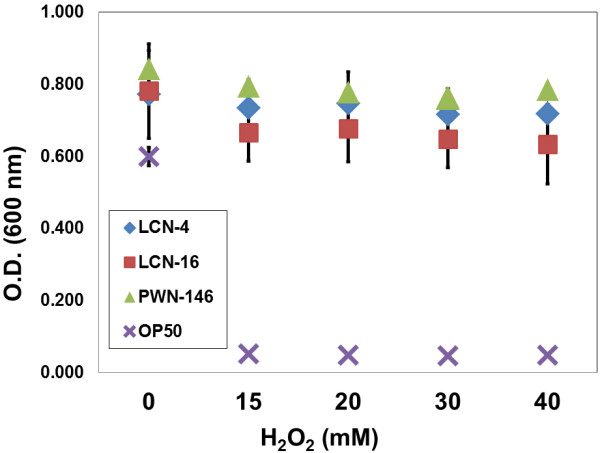
**Three *Bursaphelenchus xylophilus*-associated bacteria (*Serratia *spp. LCN-4, LCN-16 and PWN-146) have strong resistance against the oxidative stress by H**_**2**_**O**_**2**_**.** Average ± S.E. are from 3 biological replications composed of 3 technical replicate. There is no significant difference within the *Serratia* spp., but between *Serratia* spp. and *E. coli* OP50 (*p* < 0.05). Control *E. coli* OP50 could not survive under strong oxidative stress conditions.

Firstly, we examined the OS resistance of three *B. xylophilus*-associated bacteria (*Serratia* spp. LCN-4, LCN-16 and PWN-146) [8] and a control *E. coli* strain, OP50. Compared to the control strain, all three *Serratia* spp. were shown to comparably tolerate different concentrations of H_2_O_2_ ranging from 15 to 40 mM, (Figure 1). Moreover, the three isolates were able to survive up to 100 mM H_2_O_2,_ (data not shown).

**Figure 2:**
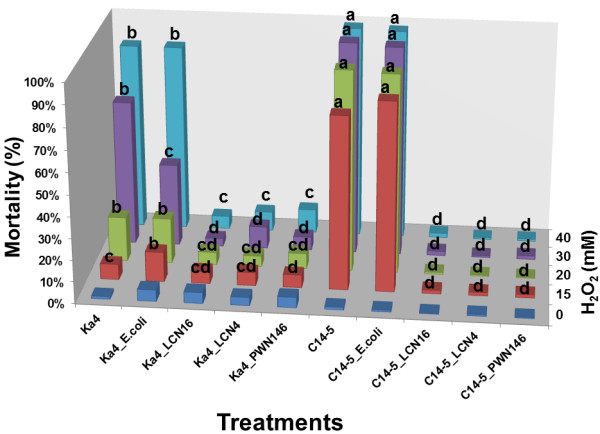
**Mortality percentages of *Bursaphelenchus xylophilus *virulent (Ka4) and avirulent (C14-5), with and without bacteria (*Serratia *spp. LCN-4, LCN-16 and PWN-146, and *E. coli *OP50) under oxidative stress conditions.** For each H_2_O_2_ condition, columns with different letters reflect statistical differences (*p* < 0.05). In control conditions (0 mM H_2_O_2_), no statistical differences were found between all treatments.

Next, we examined the OS resistance of the two *B. xylophilus* isolates with and without bacteria (Figure 2). In the absence of bacteria (surface-sterilized nematode), *B. xylophilus* isolates Ka4 (virulent) are more resistant to OS than the C14-5 (avirulent) (*p* < 0.05). At 15 and 20 mM, *B. xylophilus* Ka4 presented 73% less mortality than *B. xylophilus* C14-5. The difference of their mortality was 32% and 12% in 30 and 40 mM H_2_O_2_. To test the effect of bacteria on *B. xylophilus* survival under these conditions, we treated *B. xylophilus* with *Serratia* spp. (isolates LCN-4, LCN-16 and PWN-146) and *E. coli* OP50 for 1 h, washed away bacteria by excess and measured their OS resistance. In the presence of *Serratia* spp., both Ka4 and C14-5 were able to survive at all H_2_O_2_ concentrations tested, with mortality rates lower than 10%. Similar to the previous results of *Serratia* spp. under the OS conditions (Figure 1), there was no significant difference between the OS treatments of three bacterial isolates in association with *B. xylophilus* (*p* > 0.05). *Serratia* spp. PWN-146 was selected for further experiments. In the presence of the *E. coli* OP50, the mortality of the avirulent C14-5 isolate was higher and similar to that in nematode alone conditions (*p* > 0.05). For virulent Ka4, association with the control strain lead to similar results at 40 mM H_2_O_2_. At 30 mM H_2_O_2_, there was a significant difference with Ka4 alone (68%), with control OP50 (38%), although under the same oxidant conditions, survival of *E. coli* OP50 was significantly reduced (Figure 2). Under the other H_2_O_2_ conditions, treatment Ka4 in association with OP50 was almost similar to Ka4 alone. In non-stress conditions, all treatments were statistically equal, indicating that the bacteria used were not harmful to the nematodes.

### Observation of the nematode-bacteria association

**Figure 3:**
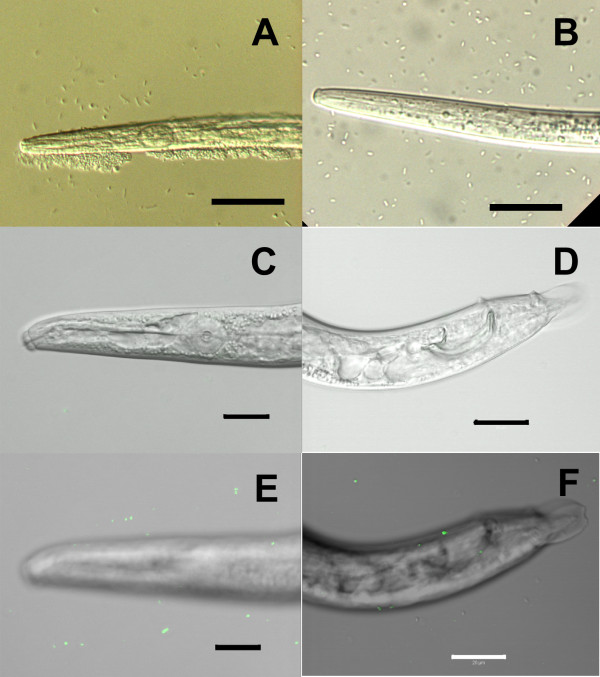
**Observation of *Serratia *sp. LCN-16 in association with *Bursaphelenchus xylophilus *after 1 h and 24 h contact. (A, B)** Differential interference contrast (DIC) microscope images of *B. xylophilus*, treated by 1 h contact of bacteria before **(A)** and after **(B)** washing with sterile DW. **(C-F)** DIC and fluorescence-merged images of *B. xylophilus*, treated by 24 h contact of bacteria and washed with sterile DW. The images of the head **(C)** and tail **(D)** region were captured in a single focal plane . Serial-section images were acquired and stacked, showing surfaces of the head **(E)** and tail **(F)** region. Scale bars, **(A)**, **(B)**, 30 μm; **(C)-(F)**, 20 μm.

After 1 h contact between *B. xylophilus* and its associated bacteria, microcolonies were found along the nematode body (Figure 3A). After extensive washing, bacteria were still present in lesser amounts, and scarcely attached to the nematode cuticle (Figure 3B). In order to test if the bacterial adhesion to the nematode became stronger, and if the nematode could uptake bacteria into its body, we performed co-culturing of the nematodes with the GFP-labelled bacteria on the same plate for 24 h. Successful GFP-labelling of *B. xylophilus*-associated bacteria was only obtained for *Serratia* spp. LCN-4 and *Serratia* spp. LCN-16. *Serratia* spp. PWN-146 were previously found to be multi-drug resistant to the antibiotics available to select for GFP-containing minitransposons [8]. After 24 h contact with *Serratia* spp. LCN-16, the density of nematode-attached bacteria was sparse (Figure 3C-F), and also no GFP fluorescence signal was detected in the nematode (Figure 3C-F). Taken together, the adhesion of these bacteria to the nematode surface and organs seems to be weak and non-specific.

### Relative gene expression of *Bxy-ctl-1* and *Bxy-ctl-2*

Using the *C. elegans* catalases (Ce-CTL-1, Ce-CTL-2 and Ce-CTL-3) as the search queries, only two catalases were predicted in the *B. xylophilus* genome, *Bxy*-CTL-1 (BUX.s00579.159) and *Bxy*-CTL-2 (BUX.s01109.377) [30]. Both cDNA sequences presented open reading frames (ORF). The longest ORF for *Bxy-ctl-1* encodes a 513 aa protein with the molecular weight of ~59kDa. The cDNA to sequence of *Bxy-ctl-2* encoded a 512 aa protein with the molecular weight of ~ 55 kDa. Both *Bxy*-CTL-1 and *Bxy*-CTL-2 were predicted as non-secretory peroxisomal proteins. However, according to Shinya *et al.*[31], *Bxy*-CTL-2 was secreted after pine wood extract stimulation. BlastP search for both catalases retrieved very similar orthologous catalases (62-64% maximum identity and *e*-value 0.0) from different species of *Caenorhabditis* and other animal parasitic nematodes, suggesting the catalases are conserved among the phylum Nematoda (Additional file 1: Figure S1 and Additional file 2: Figure S2).

**Figure 4:**
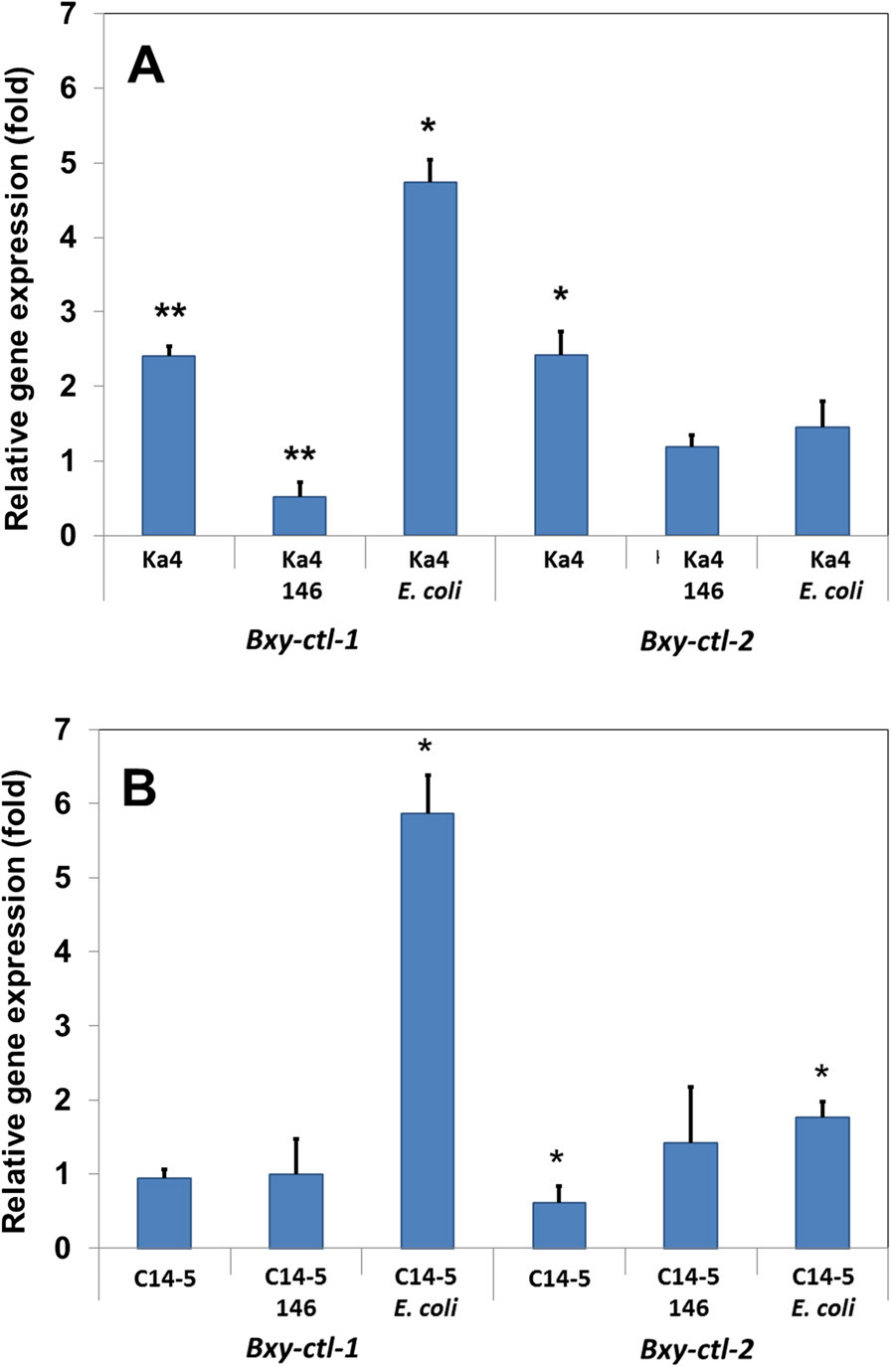
**Relative gene expression changes of *Bxy-ctl-1 *and *Bxy-ctl-2 *H**_**2**_**O**_**2 **_**treatment for 24 h.***Bursaphelenchus xylophilus* Ka4 (virulent) and C14-5 (avirulent) with and without bacteria **(A and B)** (*Serratia* spp. PWN-146 and *E. coli* OP50). *p < 0.05; **p < 0.01, compared to a normalized value of 1.00 for control nematode without H_2_O_2_.

The relative gene expression of catalase genes of *B. xylophilus* Ka4 and C14-5 with or without *Serratia* spp. PWN-146 was studied under stress conditions (Figure 4). After 24 h exposure to 15 mM H_2_O_2_, the expression levels of *Bxy-ctl-1* and *Bxy-ctl-2* genes in the *B. xylophilus* Ka4 and C14-5 were measured (Figure 4A and 4B). While virulent Ka4 catalases (*Bxy-ctl-1* and *Bxy-ctl-2*) were significantly (*p* < 0.05 and *p* < 0.01, respectively) up-regulated by nearly 2-2.5-fold compared to the non-stress condition (Figure 4A) The expression of *Bxy-ctl-1* in the avirulent C14-5 was unchanged and the expression of *Bxy-ctl-2* was slightly reduced (*p* < 0.05) (Figure 4B). These results seem to support the observations denoted in Figure 2. In the presence of the associated bacteria *Serratia* spp. PWN-146, the relative expression of Ka4 *Bxy-ctl-1* was highly suppressed (*p* < 0.01), nearly 0.5-fold less than under non-stress conditions. Under the same conditions, Ka4 expression of *Bxy-ctl-2* was not affected. The expression levels of both catalases in the avirulent C14-5 showed no significant induction or suppression. In the presence of control strain *E. coli* OP50, the expression level of *Bxy-ctl-1* in the Ka4 was induced four-fold under stress conditions, and *Bxy-ctl-2* expression level remained unchanged under non-stress conditions. Similar result was obtained for C14-5, in which *E. coli* OP50 induced 5 times more *Bxy-ctl-1* expression under stress conditions, explaining the results obtained in Figure 2. The expression levels of *Bxy-ctl-2* were also induced (*p* < 0.05), nearly 1.5-fold (Figure 4B).

## Discussion

Tolerance to host-mediated OS is an essential characteristic of plant-associated organisms. In this study, we tested if *B. xylophilus-*associated bacteria could tolerate prolonged oxidative stress conditions with or without the nematode, in an attempt to understand their behaviour in the oxidative burst conditions of the host tree in the early stages of PWD.

Plant-associated bacteria, beneficial or pathogenic, have developed efficient detoxification systems to cope with host-ROS [19,32]. This study demonstrates that *Serratia* spp. LCN-4 and LCN-16 (*S. proteamaculans*, 100% identity) and PWN-146 (*S. marcescens*, 99% identity) associated to *B. xylophilus* could sustain growth independently, and promote the survival of the nematodes under strong OS conditions. This result indicates, again, a beneficial and a potential helper effect to *B. xylophilus*. Vicente *et al*. [8] reported that some *B. xylophilus*-associated bacteria displayed plant pathogenic traits potentially related with PWD symptoms and *B. xylophilus* pathogenicity such as high cellulolytic activity, biofilm formation, EPS exudation and siderophores production. In fact, some of these traits are used by environmental bacteria as protectants against OS (i.e. EPS or biofilm). More recently, Chen *et al*. [9] showed that *B. xylophilus*-associated bacteria could support the nematode in the degradation of host xenobiotics. Based on our results, we suggest that *B. xylophilus*-associated *Serratia* spp. has evolved an elaborate detoxifying system to express several antioxidant enzymes to cope with H_2_O_2_-mediated OS.

In this study, we measured the transcript levels of two catalases in *B. xylophilus* in the presence of H_2_O_2_. PWN catalase genes presented a high protein similarity with other nematode catalases, evidencing the conserved nature of this enzyme [21]. Cap’n’collar (Cnc) transcription factors are broadly conserved in eukaryotes except for plant and fungi [33]. *C. elegans* CnC transcription factor SKN-1 regulates cellular differentiation of the pharynx and intestine during early embryogenesis, and also controls expression of many antioxidative and detoxification enzymes such as CTLs, GPXs and GSTs [34,35]. In *C. elegans* four pathways (p38 MAPK, Insulin/IGF-1 pathway, WDR-23 ubiquitin pathway, and GSK-3 pathway) are known to control SKN-1 activity and the genomic structures of these pathways are fully conserved in *B. xylophilus*[30]. Bacterial effect was transversal to virulent and avirulent *B. xylophilus.* Relative gene expression of catalase genes in *B. xylophilus* show that without bacteria, the basal expression of the both non-secreted *Bxy-ctl-1* and secreted *Bxy-ctl-2* genes in the virulent isolate Ka4, were higher than the avirulent C14-5 by 2.5-fold, which explains their differential tolerance level to H_2_O_2_. Further investigation on the detoxifying system of *B. xylophilus* is imperative. When interacting with *Serratia* spp. PWN-146, both virulent and avirulent *B. xylophilus* catalase levels decreased to levels comparable to non-stress condition, which is also in agreement with mortality test results (Figure 2).

The correlation between virulence and the ability to cope with oxidative stress has been found in the plant parasitic nematode *Melodoigyne incognita*[15,29]. Virulent *B. xylophilus* Ka4 was more tolerant to H_2_O_2_ than the avirulent *B. xylophilus* strain C14-5. Hirao *et al*. [26] reported that the susceptible *P. thunbergii* reacts to PWN invasion with a strong oxidative burst, which implies that virulent *B. xylophilus* must possess an efficient antioxidant system to cope with these conditions. Shinya *et al.*[36] suggested that potential ROS scavengers GST and GAPDH are localized on the surface coat of *B. xylophilus*. Li *et al*. [37] proposed 2-cysteine peroxiredoxin on the nematode cuticle of *B. xylophilus*, as another antioxidant agent in opposing oxidative burst. Recently, 12 anti-oxidant proteins were identified in the *B. xylophilus* secretome after plant extract stimuli, namely peroxiredoxin, catalase, glutathione peroxidase, nucleoredoxin-like protein, SOD, and thioredoxin [32]. In this context, it is essential to further investigate the possible relation between virulence of *B. xylophilus* and its tolerance to oxidative stress, which was shown for the first time in this study.

To explore the bacterial interaction with *B. xylophilus*, we have studied bacteria attachment to the nematode cuticle, an important characteristic that, to our knowledge, has not been reported before. In our experiments, the associated-bacteria were not found to strongly attach to the cuticle of *B. xylophilus*. After 24 h contact with a high concentration of GFP-tagged *Serratia* spp. LCN-16, only a few bacteria could be detected on PWN cuticle (Figure 3). Shinya *et al.*[36] have shown the presence of few bacteria on the nematode cuticle even after vigorous washing by scanning electron microscopy (SEM). *B. xylophilus* associated bacteria are reported to be carried on the nematode’s surface, and in average 290 were counted on the cuticle of PWN isolated from diseased trees [7]. If bacteria are not attached to the nematode surface, how can they be transported by *B. xylophilus* from and into a pine tree? A possible explanation could be that these bacteria are transported within the nematode [38]. However, the possible point of entry in *B. xylophilus*, the stylet opening, is very small compared with the bacteria size.

*Serratia* is an environmental ubiquitous Gram-negative bacterium, mostly free-living with an opportunistic lifestyle but also a pathogenic agent to plants, insects and humans [39]. In the plant context, *S. proteamaculans* is usually identified as an endophytic bacterium living in poplar trees [40], characterized by colonizing in harmony and even expresses PGP (plant growth promoting) traits to promote host health. *S. marcescens* is also reported as a pathogenic agent of curcubit yellow vine disease [41]. In both cases, these *Serratia* species are well adapted to the host plant (or tree) conditions, either as endophytes or pathogens, and are able to evade or suppress plant defences [42]. We could not ascertain a strong attachment of associated-*Serratia* and *B. xylophilus*. It is not unlike that these bacteria may assist the nematode in an opportunistic or facultative way, and that perhaps these bacteria could be indeed host endophytes. This hypothesis can explain why diverse bacterial communities are associated to *B. xylophilus*, and why they possess such interesting traits and host-related lifestyle. Moreover, it can help to explain the contrasting results obtained in pathogenicity tests conducted previously [8]. In this scenario, these multi-species consortia that present some *in vitro* plant-pathogenic traits that could aid the nematode inside the tree and contribute to PWD development as well [3], they could be asymptomatic endophytes that can become pathogenic as soon as the host tree is weakened [42]. Nevertheless, the host-colonizing ability of these bacteria requires further investigation.

## Conclusions

This is the first report to show that *B. xylophilus*-associated *Serratia* species can assist the nematode survival under prolonged OS conditions, revealing a possible synergism between both organisms. This beneficial effect of bacteria towards nematode resilience to OS has significant influence on PWD development. This disease is presently occurring in a variety of countries/climate zones, and might be influenced by much more various biotic and abiotic factors than previously thought.

## Methods

### *Bursaphelenchus xylophilus* isolates and culturing

Two *B. xylophilus* isolates, virulent Ka4 and avirulent C14-5 [43], were used in this study. Nematodes were cultured in *Botrytis cinerea* grown on autoclaved barley seeds at 25°C. Prior to the experiments, nematodes were extracted overnight using the Baermann funnel technique at 25°C. Nematodes were washed three times with sterilized distilled water (DW), pelleted in-between by centrifugation at 1,000 rpm during 10 min, surface cleaned with 3% L-lactic acid during 30 s, and finally washed with DW [44]. Mix-staged nematodes were used in all experiments.

### Bacteria strains and culturing

Bacterial strains and isolates used in this study are listed in Table 1. All bacteria were grown and maintained in LB plates at 28°C or 37°C (in the case of *E. coli* strains) for routine use, and in 30% (w/v) glycerol at -80°C for long-term storage. The antibiotics used in this study were: gentamycin (10–30 μg/ml), kanamycin (50 μg/ml) and ampicillin (100 μg/ml).

**Table 1:** Bacterial strains and isolates used in this study

**Bacteria used in this study**	**Genotype or Phenotype**	**Source or Reference**
*Serratia* spp. LCN-4 (100% Max. Identity: *S. proteamaculans*)	Amp^R^; Ery^R^	Bacterium associated with long lab culturing PWN. [8,45]
*Serratia* spp. LCN-16 (100% Max. Identity: *S. proteamaculans*)	Amp^R^; Ery^R^	Bacterium associated with PWN freshly isolated from wilting tree. [8,45]
*Serratia* spp. PWN-146 (99% Max. Identity: *S. marcescens*)	Amp^R^; Ery^R^; Km^R^;Tet^R^; Rif^R^	
*Escherichia coli* OP50		WormBase http://www.wormbase.org
*mini – TN7 tagging system*		
*Escherichia coli* S17::λpir (deliver)	pBK-miniTN7-gfp^2^; Gm^R^; Km^R^	[46-48]
*Escherichia coli* SM10::λpir (helper)	pUX-BF13, Amp^R^	[47]

R – resistance; Amp – ampicillin; Ery – erytromycin; Km – kanamycin; Tet – tetracyclin; Gm – gentamycin; Rif – rifampycin.

### Tagging bacteria with GFP

Bacteria (LCN-16 and PWN-146) were labelled with GFP with the vectors pBK-miniTn7-ΩGm, pBK-miniTn7-gfp2, pUX-BF13 by triparental mating method as described previously [49]. Briefly, 100 μl overnight cultures of bacteria strains and isolates (LB with appropriate antibiotics) were mixed, in 1:1:1 proportion (SM17, SM10, and LCN-16 or PWN-146), pipetted onto a 13-mm cellulose acetate filter membrane and placed on non-selective LB medium. Plates were incubated overnight at 28°C. In the following day, filters were placed into a sterile microcentrifuge tube containing 0.2 ml of 0.9% NaCl and vortexed for cell suspension. Aliquots of 100 μl of each suspension was plated onto LB with selective antibiotic (30 μg/ml gentamycin) and overnight incubated at 28°C.

### Bacteria association to nematode

Bacteria isolates (LCN-4, LCN-16 and PWN-146) and strain (OP50) were grown overnight in LB broth at 28°C or 37°C, pelleted at 10,000 rpm for 5 min, washed twice with sterilized DW, and adjusted OD_600_ for 1.00 (± 10^7^-10^8^ CFU/ml). Two approaches were used to associate bacteria with *B. xylophilus*. The first approach consisted in the observation of 1 h contact bacterial association with *B. xylophilus*, before and after washing nematodes for the oxidative stress tests. Firstly, nematodes were surface sterilized and the concentration adjusted to 150 nematodes per 50 μl of sterilized DW. Nematode-bacteria association was performed by 1 h contact between surface cleaned nematodes and 1 ml of bacterial suspension (concentrations were adjusted as described above) and in accordance to Han *et al*. [50] procedure. Afterwards, bacteria suspension was removed by pelleting the nematodes at low speed rotation (800 × g, 5 min), and then hand-picked with a nematode picker (steel wire) and transferred into a drop of sodium azide (1 M) on the centre of the agar pad [51], covered and sealed with a silicon grease-rimmed coverslip for viewing by Nomarski DIC optics.

The second approach consisted in co-culturing of *B. xylophilus* Ka4 with GFP-tagged bacteria (LCN-16-GFP; PWN-146-GFP) in 0.1% MEA plate seeded with *B. cinerea*. Firstly, nematodes were cultured on the 0.1% MEA plate for three-days, and then 500 μl of bacterial suspension (concentrations were adjusted as described above) were added and co-cultured for 24 h at 28°C. Afterwards nematodes were extracted, washed and mounted on the agar pad as described above. GFP-tagged bacteria were observed with a ZEISS Axiovert 200 microscope equipped with a confocal laser-scanning module.

### Oxidative stress tolerance tests

To test bacteria tolerance to the oxidative agent, 100 μl of freshly prepared H_2_O_2_ and 10 μl of bacteria (concentrations were adjusted as described above) were placed into each well of a 96-well plate and at a total volume of 110 μl per well. Final concentrations of H_2_O_2_ were 0, 15, 20, 30 and 40 mM. After 24 h, the plates were read in a multi-spectrophotometer (Viento, Dainippon Sumitomo Pharma, Japan) at OD_600_. For each *B. xylophilus* associated bacteria and control *E. coli*. Three independent biological replicates with three technical replicas per experiment were used for each treatment.

To test nematode and bacteria association in H_2_O_2_ oxidative conditions, first, nematodes were surface sterilized and the concentration was adjusted to 150 nematodes per 50 μl of sterilized DW, and performed 1 h nematode-bacteria association as described above. After 1 h contact with bacteria, nematodes were washed and re-suspended in sterilized DW. A 96-well plate was prepared as follows: each well received 50 μl of different H_2_O_2_ concentrations (prepared previously in double) and 50 μl of each treatment (nematode-bacteria association, nematode alone and control (DW). Three independent biological replicates with three technical replicas per experiment were used for each treatment. . Mortality of nematodes was scored after 24 h. Nematodes were considered dead, if no movements were observed after mechanical stimulation.

### Gene expression analysis of *B. xylophilus* catalases

Catalase (CTL) was selected as the antioxidant enzyme to infer gene expression differences toward the effect of H_2_O_2_ in the nematode-bacteria association. The amino acid sequences of *C. elegans* catalases (Ce-CTL-1, -2, -3) were obtained from WormBase (http://www.wormbase.org/), and used as templates for a TBLASTN search in the *B. xylophilus* Ka4 genome. The retrieved best matches were predicted as *Bxy*-CTL-1 and *Bxy*-CTL-2 of *B. xylophilus*. Predictions about general topology, domain/family, and active sites conserved were made using online tools available at Expasy WWW pages (http://www.expasy.org/tools/).

Gene expression of *Bxy-ctl-1* and *Bxy-ctl-2* were analysed by qRT-PCR using SYBR® green assay. Total RNA was extracted from 24 h-stressed nematodes (treatments: nematodes alone and nematode-bacteria association) in 15 mM H_2_O_2_, using CellAmp Direct RNA Prep Kit for RT-PCR (Real time) (Takara Bio Inc., Japan) and following manufacturer’s instructions. The concentration and quality was measured using NanoVue plus spectophotometer (GE Healthcare Life Sciences, USA). Total RNA (adjusted for concentration of 50 ng/μl) was reverse transcribed using Oligo dT primer and PrimeScript RT enzyme from PrimeScript™ RT reagent Kit (Perfect Real Time) (Takara Bio Inc., Japan). Quantitative RT-PCR was performed using CFX96™ Real-Time (Bio-Rad), and SYBR Premix Ex TaqTM II (Tli RnaseH Plus) kit (Takara Bio Inc., Japan). The housekeeping actin gene *Bxy-act-1* was used as an internal control gene for calculation of relative expression levels of each antioxidant gene [52]. Primers were designed using Prime 3 software [53] and tested for specificity prior to qPCR. The primers used for *Bxy-act-1*, *Bxy-ctl-1* and *Bxy-ctl-2* genes amplification were listed in Additional file 3: Table S1. Two independent biological replicates with two technical replicas per experiment were used for each qPCR test. No template controls (NTC) were prepared for each qPCR run. Thermal cycling conditions were: initial denaturation at 95°C for 30 sec; 39 cycles of denaturation at 95°C for 5 sec, annealing and extension at 60°C for 30°C; followed by the melting curve. A single peak at the melting temperature of the PCR-product confirmed primer specificity.

Relative gene expression of each gene were analysed using ΔΔC_T_ Method [52]. The data were analysed with Ct values in normal and stress conditions and using the following equation: ΔΔCT = (C_T,Target_ ‒ C_T,actin_)_normal_ ‒ (CT, _Target_ ‒ C_T,Actin_)_stress_. The fold change in *Bxy-ctl-1* and *Bxy-ctl-2* was normalized to *Bxy-act-1* and relative to the expression at normal conditions, was calculated for each sample using the equation above.

### Statistical analysis

Statistical analysis was performed using SPSS 11.5. Data represent the mean ± standard error (SE). Statistical significance was inferred by one-way ANOVA and post hoc multi-comparison Duncan test.

## Competing interests

The authors declare that they have no competing interests.

## Author contributions

Conceived and designed the experiments: CSLV, KH. Performed the experiments: CSLV, YI, KH. Analyzed the data: CSLV, YI, KH. Wrote the paper: CSLV, MM, KH. All authors read and approved the final manuscript.

## Supplementary Material

Additional file 1: Figure S1 Alignment of deduced amino acid sequences from catalase 1 (CTL-1) with the top matches in database. Residues conserved are highlighted in dark grey and marked by an asterisk. *Bursaphelenchus xylophilus* CTL-1; Caenorhabditis elegans CTL-1 (CAA74393.1); C. remanei CTL-3 (XP_003102502.1); C. briggsae hypothetical protein (XP_002631620.1); Ditylenchus destructor CTL (AFJ15102.1). Click here for file

Additional file 2: Figure S2 Alignment of deduced amino acid sequences from catalase 2 (CTL2) with the top matches in database. Residues conserved are highlighted in dark grey and marked by an asterisk. *Bursaphelenchus xylophilus* CTL-2; Caenorhabditis elegans CTL-3 (NP741058.1); C. brenneri CTL-2 (EGT40792.1); Haemonchus contortus CTL (AAT28330.1); Ditylenchus destructor CTL (AFJ15102.1). Click here for file

Additional file 3: Table S1 Primers used in this study. Click here for file
