# Supplementary figures and images for: Pinewood nematode-associated bacteria contribute to oxidative stress resistance of Bursaphelenchus xylophilus
Source: BMC Microbiol. 2013 Dec 23;13:299. doi: 10.1186/1471-2180-13-299 (PMC3880045; doi:10.1186/1471-2180-13-299)

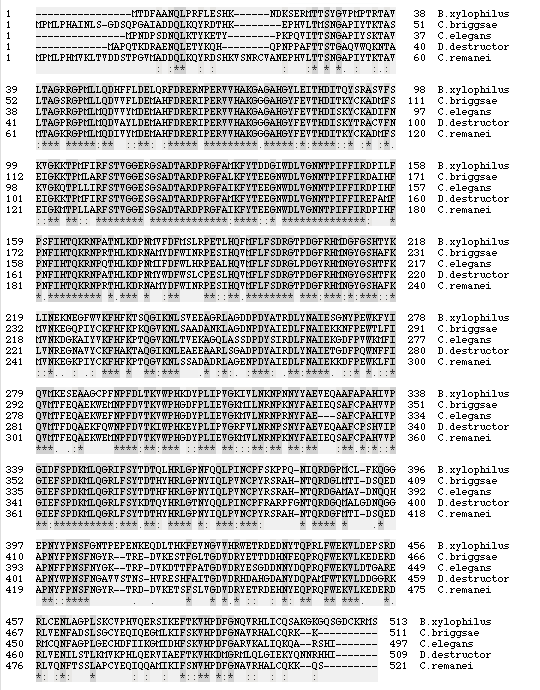


**Figure S1**

Supplement: Additional file 1: Figure S1 — Alignment of deduced amino acid sequences from catalase 1 (CTL-1) with the top matches in database. Residues conserved are highlighted in dark grey and marked by an asterisk. Bursaphelenchus xylophilus CTL-1; Caenorhabditis elegans CTL-1 (CAA74393.1); C. remanei CTL-3 (XP_003102502.1); C. briggsae hypothetical protein (XP_002631620.1); Ditylenchus destructor CTL (AFJ15102.1). [file 1471-2180-13-299-S1.doc]

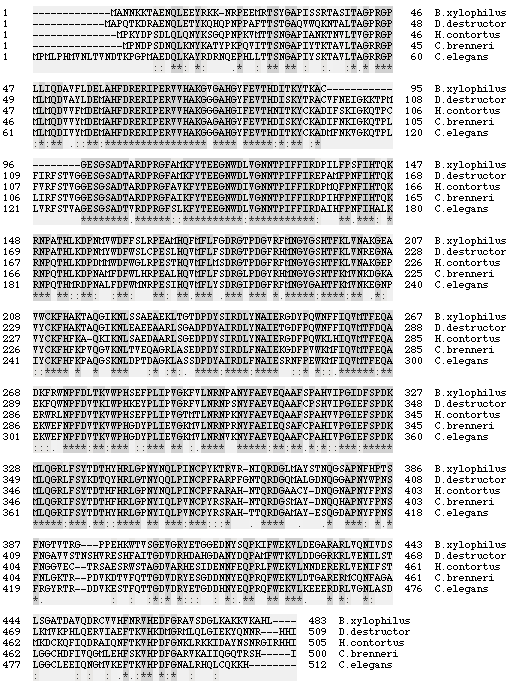


**Figure S2**

Supplement: Additional file 2: Figure S2 — Alignment of deduced amino acid sequences from catalase 2 (CTL2) with the top matches in database. Residues conserved are highlighted in dark grey and marked by an asterisk. Bursaphelenchus xylophilus CTL-2; Caenorhabditis elegans CTL-3 (NP741058.1); C. brenneri CTL-2 (EGT40792.1); Haemonchus contortus CTL (AAT28330.1); Ditylenchus destructor CTL (AFJ15102.1). [file 1471-2180-13-299-S2.doc]
