# Supplementary material for: Pinewood nematode-associated bacteria contribute to oxidative stress resistance of Bursaphelenchus xylophilus
Source: BMC Microbiol. 2013 Dec 23;13:299. doi: 10.1186/1471-2180-13-299 (PMC3880045; doi:10.1186/1471-2180-13-299)
Supplement: Additional file 3: Table S1 — Primers used in this study. [file 1471-2180-13-299-S3.doc]

**Additional file 3. Table S1**

*Bxy-act-1*for, 5’ -CATCCTCCGTCTCGACTTGG-3’

*Bxy-act-1*rev 5’-ATGTCACGCACGATTTCACG-3’

*Byx-ctl-1* for, 5’-GCCAGCGTCTTCAGCAAAGT-3’

*Bxy-ctl-1*rev, 5’-CCAAATTCCGTCATCGGTGT-3;

*Byx-ctl-2* for, 5’-CCGACTTCTTTCAACGGAAC-3’

*Bxy-ctl-2* rev, 5’-CCTTCATCGAGCACCTTTTC-3’.
